# Supplementary material for: Deletion of wfs1 Impairs Oligodendrocyte Precursor Cells Dorsal Distribution and Myelination Through the wfs1-hmgcs1 Axis in Zebrafish
Source: Biology (Basel). 2026 Aug 16;15(16):1402. doi: 10.3390/biology15161402 (PMC13510151; doi:10.3390/biology15161402)
Supplement: Supplementary file 1 [file biology-15-01402-s001.zip › File S1-Transcriptome sequencing data and differentially expressed genes (DEGs) in wfs1-- zebrafish at 5 dpf/2.KEGG/wfs1KOvsWT/GSEA/WT/DNA_REPLICATION/DNA_REPLICATION(DRE03030).html]

Details for gene set DNA\_REPLICATION(DRE03030)[GSEA]

|  || Dataset | deg.sample |
| Phenotype | sample.cls |
| Upregulated in class | WT |
| GeneSet | DNA\_REPLICATION(DRE03030) |
| Enrichment Score (ES) | -0.3109516 |
| Normalized Enrichment Score (NES) | -1.1579695 |
| Nominal p-value | 0.29554656 |
| FDR q-value | 0.54274684 |
| FWER p-Value | 1.0 |
Table: GSEA Results Summary

  

Fig 1: Enrichment plot: DNA\_REPLICATION(DRE03030)      
 Profile of the Running ES Score & Positions of GeneSet Members on the Rank Ordered List

  

| PROBE | DESCRIPTION (from dataset) | GENE SYMBOL | GENE\_TITLE | RANK IN GENE LIST | RANK METRIC SCORE | RUNNING ES | CORE ENRICHMENT || 1 | 393241\_1 | rnaseh2b |  |  | 521 | 2.921 | 0.0859 | No |
| 2 | 393774 | pole3 |  |  | 941 | 2.179 | 0.1490 | No |
| 3 | 556995\_1 | lig1 |  |  | 956 | 2.157 | 0.2241 | No |
| 4 | 559535\_1 | dna2 |  |  | 3928 | 0.843 | 0.1603 | No |
| 5 | 564982\_2 | mcm6l |  |  | 4088 | 0.814 | 0.1838 | No |
| 6 | 556456\_1 | pold1 |  |  | 4702 | 0.718 | 0.1897 | No |
| 7 | 100037336 | rfc1 |  |  | 5563 | 0.631 | 0.1848 | No |
| 8 | 286747 | mcm5 |  |  | 8517 | 0.401 | 0.1061 | No |
| 9 | 337598 | mcm4 |  |  | 8592 | 0.396 | 0.1177 | No |
| 10 | 259256\_1 | rfc3 |  |  | 8868 | 0.377 | 0.1222 | No |
| 11 | 317740 | pola1 |  |  | 9215 | 0.352 | 0.1237 | No |
| 12 | 259256 | rfc3 |  |  | 9590 | 0.327 | 0.1234 | No |
| 13 | 566409 | mcm6 |  |  | 9603 | 0.327 | 0.1345 | No |
| 14 | 192323 | mcm3 |  |  | 9643 | 0.324 | 0.1446 | No |
| 15 | 407653 | prim2 |  |  | 9900 | 0.310 | 0.1474 | No |
| 16 | 65226 | rpa2 |  |  | 10674 | 0.262 | 0.1323 | No |
| 17 | 192320 | pole2 |  |  | 10741 | 0.259 | 0.1393 | No |
| 18 | 406435 | rfc4 |  |  | 10835 | 0.253 | 0.1452 | No |
| 19 | 692256 | pold3 |  |  | 11051 | 0.241 | 0.1469 | No |
| 20 | 100005387 | rnaseh2c |  |  | 11399 | 0.222 | 0.1438 | No |
| 21 | 100147915 | mcm3l |  |  | 11619 | 0.210 | 0.1442 | No |
| 22 | 30440 | prim1 |  |  | 12624 | 0.158 | 0.1182 | No |
| 23 | 556456 | pold1 |  |  | 13726 | 0.106 | 0.0874 | No |
| 24 | 796456 | pold2 |  |  | 13771 | 0.103 | 0.0896 | No |
| 25 | 192338 | mcm2 |  |  | 14301 | 0.078 | 0.0757 | No |
| 26 | 556995 | lig1 |  |  | 14356 | 0.076 | 0.0767 | No |
| 27 | 322059 | pola2 |  |  | 14360 | 0.076 | 0.0792 | No |
| 28 | 386707 | fen1 |  |  | 14956 | 0.049 | 0.0623 | No |
| 29 | 393196 | rpa3 |  |  | 16744 | -0.024 | 0.0070 | No |
| 30 | 571834 | si:dkey-28b4.7 |  |  | 16805 | -0.027 | 0.0060 | No |
| 31 | 445385 | rfc5 |  |  | 16927 | -0.032 | 0.0033 | No |
| 32 | 553610 | pole4 |  |  | 17453 | -0.052 | -0.0113 | No |
| 33 | 550274 | zgc:110269 |  |  | 17476 | -0.053 | -0.0102 | No |
| 34 | 327491 | rpa1 |  |  | 17594 | -0.058 | -0.0118 | No |
| 35 | 192333 | mcm7 |  |  | 18162 | -0.084 | -0.0267 | No |
| 36 | 553405 | pole |  |  | 18294 | -0.089 | -0.0277 | No |
| 37 | 30678 | pcna |  |  | 18983 | -0.121 | -0.0451 | No |
| 38 | 393241 | rnaseh2b |  |  | 20031 | -0.168 | -0.0721 | No |
| 39 | 503748 | rfc2 |  |  | 22293 | -0.284 | -0.1332 | No |
| 40 | 393195 | rnaseh2a |  |  | 22555 | -0.299 | -0.1309 | No |
| 41 | 550504 | ssbp1 |  |  | 22792 | -0.313 | -0.1274 | No |
| 42 | 436932 | rnaseh1 |  |  | 24127 | -0.401 | -0.1552 | No |
| 43 | 564982\_1 | mcm6l |  |  | 25025 | -0.448 | -0.1677 | No |
| 44 | 559535 | dna2 |  |  | 27848 | -0.722 | -0.2311 | No |
| 45 | 553405\_1 | pole |  |  | 30393 | -1.444 | -0.2604 | Yes |
| 46 | 556995\_2 | lig1 |  |  | 30840 | -1.799 | -0.2114 | Yes |
| 47 | 393241\_2 | rnaseh2b |  |  | 31110 | -2.106 | -0.1461 | Yes |
| 48 | 393774\_2 | pole3 |  |  | 31836 | -4.872 | 0.0017 | Yes |
Table: GSEA details [plain text format]

  

Fig 2: DNA\_REPLICATION(DRE03030)      
 Blue-Pink O' Gram in the Space of the Analyzed GeneSet

  

Fig 3: DNA\_REPLICATION(DRE03030): Random ES distribution      
 Gene set null distribution of ES for **DNA\_REPLICATION(DRE03030)**
